# In-vitro antioxidant potential and acetylcholinesterase inhibitory effect of Ficus benghalensis aerial root extract

**DOI:** 10.4314/ahs.v22i4.34

**Published:** 2022-12

**Authors:** Anand Ramasamy, K Anandakumar, K Kathiresan

**Affiliations:** 1 Research Scholar, Department of Pharmacy, Annamalai University, Annamali Nagar, Tamil Nadu, India; 2 Professor and Head, Department of Pharmaceutical Analysis, Swamy Vivekanandha College of Pharmacy, Elayampalayam, Tamil Nadu, India; 3 Associate Professor, Department of Pharmacy, Annamalai University, Annamali Nagar, Tamil Nadu, India

**Keywords:** *Ficus benghalensis*, antioxidant, DPPH, acetylcholinesterase, neurodegenerative disorder

## Abstract

**Aim and objective:**

The aim of the current study was to evaluate the antioxidant effect, acetylcholinesterase (AChE) inhibitory effect and phytochemical screening of different extracts of aerial root extract of *Ficus benghalensis using in-vitro* methods.

**Methods:**

The aerial root extract was prepared by successive extraction method using different organic solvents having increasing order of polarity. FB aerial root extract was screened for preliminary phytochemical analysis. FB aerial root extracts were evaluated for in-vitro acetylcholinesterase inhibitory effect by the Ellman's method and anti-oxidant potential by DPPH assay and hydroxyl radical neutralizing assay.

**Results:**

Preliminary phytochemical screening of FB extracts indicate the existence of the phytochemicals such as phenols, alkaloids, flavonoids, glycosides, anthraquinones, tannins and steroids. The results of the DPPH assay, hydroxyl radical scavenging assay and AChE inhibitory assay show that chloroform and ethyl acetate extracts are having significant antioxidant activity and acetylcholinesterase inhibitory effect as compared to the other extracts, respectively.

**Conclusion:**

The results of the current study suggest that the aerial root extract of FB might be a potential drug source for treatment of neurodegenerative disorders like Alzheimer disease.

## Introduction

Oxygen is an essential element for life. The cells generate energy in the form of adenosine triphosphate in mitochondria by using oxygen, during this process free radicals are generated as a by-product. The free radicals generated during this energy synthesis process are mainly reactive oxygen species (ROS) and reactive nitrogen species (RNS). The above free radicals have both physiological and deleterious roles.^1^–^3^

The term free radicals include the reactive species, ROS and RNS, and the other non-radical reactive molecules are known as oxidants. The free radicals are having strong reactivity, but they are less stable than the oxidants.^4^,^5^

Superoxide (O2-.), hydroxyl radical (OH.) and nitric monoxide (NO.) are the general cellular free radicals. The hydrogen peroxide (H2O2) and peroxynitrites (ONOO-) are non-radicals even though they generate free radicals through various biochemical processes.^6^

The formation ROS and RNS inside the cells are by means of enzymatic and non-enzymatic process. At normal range, ROS and RNS involved in the cell structure maturation process and act as a weapon for the host immune system.^7^ Other beneficial effects of ROS and RNS are involved in various cell signalling, for e.g., NO functions as a secondary messenger intercellular level and alters the blood vessel diameter, inhibits thrombus formation, and regulate neuronal function^8^,^9^

Antioxidants is a defence system present in our body to counteract oxidative damage from oxygen free radical generated through aerobic metabolism.^10^ Endogenous and exogenous antioxidants, such as citrulline, arginine, glutathione, taurine, selenium, creatine, zinc, vitamin (A, E & C) and substances like superoxide dismutase (SOD), catalase, glutathione reductase, and glutathione peroxidase were the enzymes which are antioxidants helps to regulate the ROS and RNS generated.^11^–^13^

We were frequently exposed to the free radicals generated from the man-made environmental pollutants such as smoke, radiation and metal ions. Over accumulation of free radicals and non radical species will damage the cellular lipids, proteins, DNA and also inhibit their normal physiological function. In recent days, oxidative stress was the major cause for various human diseases including the ageing process. Oxidative stress is characterized by an increase of the levels of free radicals or inability to neutralize the free radicals by the antioxidants.^14^,^15^

Many studies show that chronic oxidative stress and neuroinflammation are major factors responsible for the progressive neurodegeneration in the CNS disorders.^16^,^17^ In CNS disorders, Alzheimer disease (AD) is a major neurodegenerative disorder and commonly causes cognitive defects in aged people^18^. Many studies have shown that the ROS damage was found in the particular regions of brain where selective neurodegeneration happens in AD. The lipid per oxidation indicators 4-hydroxynonenal and malondialdehyde are found in the cerebral cortex and hippocampus regions of AD patients.^19^–^21^ AD is associated with the formation of neuro fibrillary tangles which are composed of Tau protein, senile plaques made up of amyloid beta protein and there is substantial loss of cholinergic and cortical neurons.^22^,^23^ Many evidence shows that loss of acetylcholine a cholinergic neurotransmitter is responsible for the cognitive impairment. In the cerebral cortex within the synapses the Acetylcholine was degraded by the enzyme Acetylcholinesterase (AChE).^24^, ^25^ At present there is no drug which completely cures AD. Currently available AD dugs are mainly used to manage the symptoms of AD, for example at present the acetylcholine esterase inhibitors were currently used to manage the cognitive deficits of AD.^26^ Various studies show that treatment with antioxidants reduced the toxic effects of β-amyloid and improve cognitive function in experimental animals.^27^–^31^ So, AChE and oxidative stress might be attractive target for the drug discovery for AD.

FB belongs to the Moraceae family is commonly distributed in India. FB has been used in Indian ancient medicine to manage various ailments including Central nervous system disorders.^32^ Our objective was to evaluate the antioxidant effect, AChE inhibitory activity and phytochemical screening of different extracts of aerial root of Ficus benghalensis.

## Experimental Methods

### Chemicals

Acetyl thiocholine iodide, 1,1-Di-phenyl -2-picrylhydrazyl (DPPH), cholinesterase, 5,5′-dithiobis (2-nitro benzoic acid) (DTNB), Ascorbic Acid and Methanol and all other chemicals are procured from Sigma Aldrich and Fischer Scientific. Donepezil was obtained as a gift sample from a pharmaceutical company in Ahmedabad, Gujarat.

### Collection of plant

Fresh aerial roots of *Ficus benghalensi* aerial roots were collected from the rural area of Namakkal district located at Tamil Nadu, India. The FB aerial root was taxonomically identified and authenticated and a voucher of aerial root specimen has been deposited at the department herbarium.

### Preparation of Extract

The aerial root extract was prepared by consecutive extraction method using different organic solvents like Pet ether, Chloroform, Ethyl acetate, Acetone, Methanol and Water (1:10 w/v) having polarity in an increased order. The aerial root powder (200 g) was packed in the Soxhlet apparatus and in the round bottomed flask petroleum ether (2 litres) was added and refluxed for 7 h. Then the aerial root extract was dried by using vacuum rotary evaporator. Then the dried successive petroleum ether extract (yield 2.0153%) was stored at 4°C for further study. Petroleum ether extracted plant material was completely dried and subjected to further successive extraction by using Soxhlet apparatus with various solvents as mentioned below in an increasing polarity manner. The extracts were dried and stored as mentioned above.

- Ficus benghalensis Pet Ether Extract = FPE
- Ficus benghalensis Chloroform Extract = FCE
- Ficus benghalensis Ethyl acetate Extract = FEE
- Ficus benghalensis Acetone Extract = FAE
- Ficus benghalensis Methanol Extract = FME
- Ficus benghalensis Water Extract = FWE

### Phytochemical Screening

The qualitative phytochemicals screening of aerial root extracts of *Ficus benghalensis* were carried out using standard procedures for determination of total alkaloids, flavonoids, phenolic compounds, steroids, tannins, saponins, Terpenoids and glycosides.^33^

### Anti-oxidant Assay

#### DPPH Assay

% Inhibition = (C_A_ − T_A_)/C_A_ x 100

FB aerial root extracts were assessed for free radical neutralizing activity by using DPPH assay method.^34^ Different concentrations of Ficus benghalensis aerial root extracts (20–100 µg/ml) were treated with 0.1 mM DPPH, after the vigorous shaking the mixture were kept for 30 mins at 27oC after that the absorbance was taken at 517 nm. The absorbance measured in this assay was inversely proportional to the free radical scavenging activity. Ascorbic acid (10–100 mg/ml) has been served as a standard antioxidant. Percentage (%) inhibition was estimated by the below formula.

CA= absorbance of control, TA = absorbance of test.

The concentration showing 50% of inhibition (IC50) was estimated from the curve plotted with different concentrations of inhibition.

### Hydroxyl Radical Assay

In this study, the hydroxyl free radical scavenging effect of FB extracts were estimated by the method described by Shelley M Klein.^35^ The principle of this method was the estimation of the product obtained from the breakdown of 2-deoxy-D-ribose by thiobarbituric acid. Hydroxyl free radical has been produced by the Fenton reaction. Make the test solution volume to 1 ml with 2.8 mM of 2- deoxy- D-ribose, 20 mM of potassium dihydrogen phosphate buffer, 100 µM of ferric chloride, 100 µM of EDTA, 1 mM of H2O2, 100 µM Ascorbic acid, to which different range of FB aerial root extracts or standard samples (20–100 µg/ml) were added. After keeping the mixture to stand for 1 h at 37 °C to the 0.5 ml of the above solution add 1 ml of 2.8 % CCl3COOH and 1 ml of 1% aqueous Thio barbituric acid, then keep the mixtures for 15 mins at 90 °C to produce the colour. Allow the mixture to cool, and then absorbance was taken at 532 nm with the respective blank solution. Percentage (%) inhibition was estimated by the below formula

% Inhibition = (C_A_ − T_A_)/C_A_ x 100

CA= absorbance of control, TA = absorbance of test.

The concentration showing 50% of inhibition (IC50) was estimated from the curve plotted with different concentrations of inhibition.

### Estimation of in- vitro Acetylcholinesterase (AChE) Inhibition

% Inhibition = (C_A_ − T_A_)/C_A_ x 100

Acetylcholinesterase inhibitory effect was estimated by using the method described by Ellman.^36^ The AChE hydrolyses the acetylthiocholine to thiocholine and interacts with 5,5′-dithiobis (2-nitro benzoic acid) (Ellman reagent) and forms 2-nitro benzoate -5-mercapto thio choline and 5 -thio -2-nitro benzoate and the absorbance was taken at 412 nm. Tris-HCl buffer (50 mM, pH 8) was used in the experiment. The acetylcholine enzyme was diluted 0.1% Bovine Serum Albumin. Ellman's reagent was dissolved in the Tris-HCl buffer consists of 0.1M sodium chloride and 0.02 M magnesium chloride. Acetylthiocholine iodide was dissolved in the deionized water. Ellman's reagent (3mM, 100 µl), Acetylcholinesterase (0.26 U/ml, 20 µl) and TRIS-HCL buffer (40 µl), 20 µl FB aerial root extract in a concentration (20 - 100 µg/ml) mixed in buffer containing 10% methanol are added in the 96-well plates. After mixing, the microplate was kept at 25oC for 15 min after that absorbance taken at 412 nm by using microplate reader and kept as blank values. The enzyme inhibition reaction was induced by adding of 20 µl of 15 mM acetylthiocholine iodide, the acetylcholine undergoes hydrolysis which was estimated by measuring the absorbance at 412 nm for every 5 min till 20 min. Donepezil served as reference drug. Percentage (%) of enzyme inhibition was estimated by using below formula

CA= absorbance of control, TA = absorbance of test.

The concentration showing 50% of inhibition (IC50) was estimated from the curve plotted with different concentrations of inhibition.

### Statistical Analysis

One way analysis of variant then the Tukey's multiple comparison test are done to estimate measurable differences in between the test and Standard groups. Results were presented as mean ± standard error of mean.

## Results

### FB Extraction Yield and Phytochemical Screening

The percentage yield of Ficus benghalensis with different solvent extracts was listed in the Table.1. Extraction with Petroleum Ether, Methanol and Water showed highest percentage yield of 2.015%, 1.74%, 1.65% respectively, as compared to other solvents extracts.

**Table 1:** Percentage yield Ficus benghalensis with different solvent extracts

S.No	Solvents used for successive Extraction	% Yield of the Extract(w/w)
**1**	Petroleum Ether	2.0151
**2**	Chloroform	0.5923
**3**	Ethyl Acetate	0.6443
**4**	Acetone	0.3703
**5**	Methanol	1.7421
**6**	Water	1.6521

The qualitative determinations of phytochemicals present in the different solvent extracts are presented in the Table.2. Result showed Flavanoid, Glycosides, sterols, reducing sugars, Phenols and triterpenes were present in the different extracts of aerial root of Ficus benghalensis. The phytochemicals found in the present study are k nown to have medicinal properties.^37^, ^38^

**Table 2:** Preliminary Phytochemical Constituents of *Ficus benghalensis*

Name of the Compound	Identification Test	Different Extract of aerial root of *Ficus benghalensis*					
		FPF	FCF	FEF	FAF	FMF	FWF
Alkaloids	Dragendroff and Wagners Test	-	+	-	-	+	+
Flavanoids	Shinoda test	-	+	+	-	+	-
Phenol	Ferric Chloride Test	-	-	+	+	+	-
Saponins	Persistent foam tests	-	-	-	-	-	-
Steroid	Acetic anhydride test	+	+	+	-	-	-
Terpenoid	Salkowski test	-	-	-	-	-	-
Anthraquinones	Borntrager test	-	-	+	-	-	+
Cardiac Glycosides	Keller killani test	-	+	+	-	+	+
Tannins	Ferric Chloride Test	-	-	+	-	-	-

+ Positive = present, - Negative = Absent

### In-vitro Antioxidant Activity

**Figure 1:**
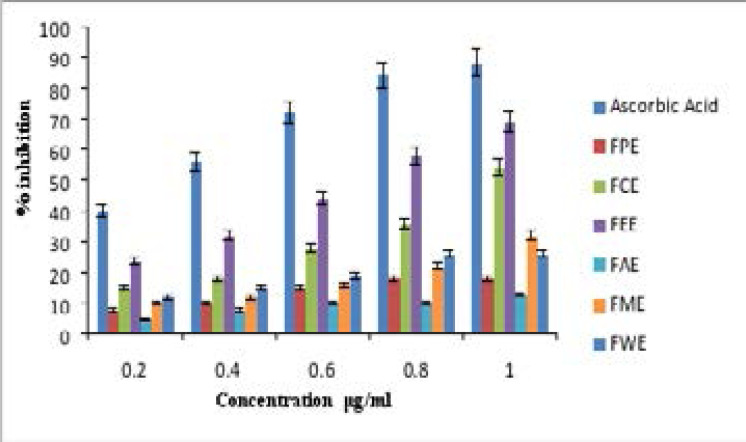
DPPH assay percentage of Inhibition

Various studies have strongly implicated that oxidative stress-mediated damage was major response for the pathogenesis of various neurodegenerative diseases including AD. Antioxidants will regulate the oxidative stress in the biological system and exhibits neuroprotective effects. 39 The antioxidant effect of FB aerial root extracts was assessed by DPPH and Hydroxyl free radical assay. In the DPPH assay the percentage free radical (%) inhibition of Ethyl acetate and Chloroform extract of FB were comparable to the standard antioxidant Ascorbic acid which was represented in Figure 1. The IC_50_ values of the DPPH assay are presented in Table 3. Ethyl acetate and chloroform extract having an IC_50_ of 68±0.06 µg/ml, 101.2±0.36 µg/ml respectively and Ascorbic acid with IC_50_ of 30.09±0.03 µg/ml.

**Table 3:** IC_50_ Value of DPPH Assay, Hydroxy Free radical Scavenging Assay

Assay Method	DPPH(IC_50_) µg/ml	Hydroxyl Radical Scavenging (IC_50_) µg/ml
**FPF**	318.4±0.31	242.6±0.21
**FCF**	101.2±0.36	93±0.27
**FEF**	68±0.06	76.2±0.12
**FAF**	512±0.06	642.8±0.34
**FMF**	177±0.41	141.6±0.32
**FWF**	215.8±0.09	197.8±0.21
**Ascorbic Acid**	30.09±0.03	44.6±0.05

All the results were average of triplicate and presented as mean ± standard deviation

**Figure 2:**
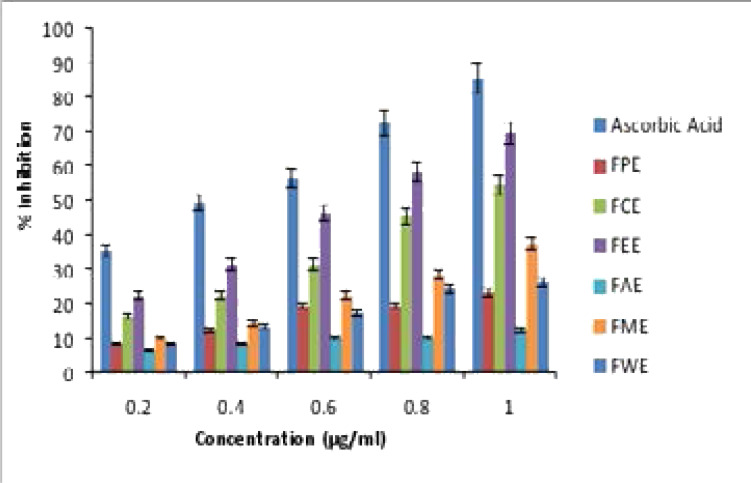
Hydroxyl Radical assay percentage of Inhibition

In the Hydroxyl free radical neutralizing assay also, the percentage of inhibition was good in Ethyl acetate and Chloroform FB extract as comparable to Ascorbic acid which was represented in Figure.2. The IC_50_ values presented in Table.3 shows that IC_50_ of Ethyl acetate was 76.2±0.12 µg/ml, Chloroform was 93±0.27 µg/ml and the IC_50_ value of Ascorbic acid was 44.6±0.05 µg/ml for the hydroxyl radical neutralizing assay.

In both the anti-oxidant assay, the Ethyl acetate and Chloroform FB extract shows comparable effect with that of standard Ascorbic acid. And all other extracts showing decreased percentage of inhibition with high IC50 values.

### In-vitro Acetyl cholinesterase (AChE) Inhibitory Activity

**Figure 3:**
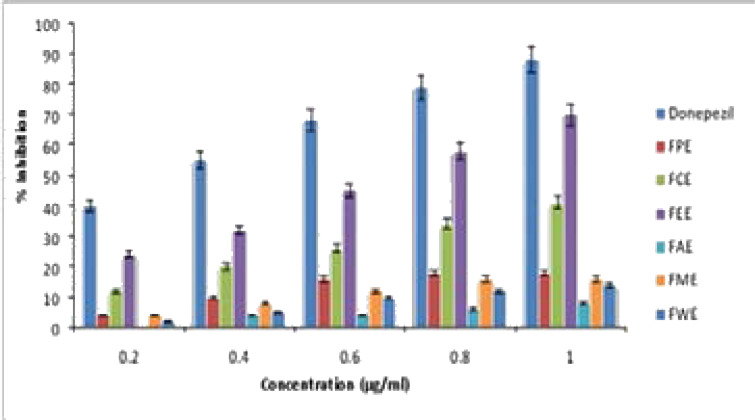
Acetylcholinesterase (AChE) Inhibitory Activity

Experimental evidences illustrate cholinesterase inhibition has become the promising strategy to treating the symptoms of AD. AChE accelerates Aβ peptide aggregation and leads to the Aβ -AChE complex formation at the synaptic region of hippocampus leading to pathogenesis AD.^40^ In this study we investigated the inhibitory effect against AChE with various extracts of Ficus benghalensis. Among all the extracts only Ethyl acetate extract was showing comparable percentage of inhibition with that of standard Donepezil which was shown in Figure 3. IC50 value of Ethyl acetate was 67±0.06 µg/ml and Donepezil was 33.20±0.23 µg/ml. Except Ethyl acetate all other extracts showed less Ache inhibition with high IC_50_ value.

## Discussion

Alzheimer's disease (AD) is one of the leading neurodegenerative disorders characterized with memory impairment, cognition and behaviour deficits, but the molecular mechanism of which is not yet known. There is no drug which is completely protects the neuron. There are two general conceptual approaches in the discovery of new drugs for the treatment of AD. The first one is preventing the onset of the disease by attenuating the primary factors which reduces the secondary pathological cause of the disease and slows down the disease progression. Second one is symptomatic treatment that treats the cognitive deficits of the disease and protects from further cognitive impairment. But the rational treatment strategies depend upon the seriousness of the disease and the condition of the patients. Present therapeutic agents like Acetyl cholinesterase inhibitors are mainly targeting the specific symptoms such as improvement of cholinergic neurotransmission and inhibit the breakdown of acetylcholine within the synapse. And the other treatment agents for AD remains controversial. 41,42

However, the acetyl cholinesterase inhibitors used at present for the symptomatic treatment are very less and having their own limitations. 43 Even chemical antioxidants used were also produce adverse effects like, hepatotoxic and carcinogenesis.^44^. Accumulating evidence suggests that the natural products derived bioactive compounds as one of the major sources for neuroprotection. Even the cholinesterase inhibitor galantamine is a natural product and rivastigmine is a semi-synthetic compound derived from the natural product called physostigmine.^45^

Traditionally, different plant parts of Ficus benghalensis are claimed to posses immunomodulatory^46^ hypoglycemic, 47 antioxidant,^48^ antistress & antiallergic,^49^ Analgesic,^50^ anthelmintic^51^ and Wound-healing activities.^52^ There are not much studies on the aerial root of Ficus benghalensis in the neuroprotection. In this study the antioxidant effect of FB aerial root extracts was estimated by DPPH assay and Hydroxyl radical scavenging assay shows that the Ethyl acetate and Chloroform extract having comparable antioxidant effect with that of standard Ascorbic acid. We also estimated the AChE inhibitory effect of the various aerial root extracts of Ficus benghalensis, it also shows that Ethyl acetate extract showed comparable effect with that of standard Donepezil. Hence the result suggest that the presence of free radical neutralization and AChE inhibition of Ethyl acetate and Chloroform extract may be due to the existence of various Phytochemicals in the FB aerial root extracts.

## Conclusion

Aerial root extract of Ficus benghalensis demonstrated good antioxidant with AChE inhibitory effect. Further research is required to explore the active phytochemical compound along with their mechanism of action to aid the discovery of new drugs for the treatment of Alzheimer Disease.

## Figures and Tables

**Table 4:** IC_50_ Value of Acetyl cholinesterase (AChE) Inhibitory Activity

Assay Method	Acetylcholinesterase (AChE) inhibitory activity (IC_50_) µg/ml
**FPF**	264.2± 0.32
**FCF**	132.6±0.21
**FEF**	67±0.06
**FAF**	546±0.21
**FMF**	302±0.06
**FWF**	318±0.45
**Donepezil**	33.20±0.23

All the results were average of triplicate and presented as mean ± standard deviation
